# Postoperative Intravenous Fluids and Electrolytes Management After Gastrointestinal Surgery in a Sudanese Teaching Hospital: A Prospective Audit

**DOI:** 10.7759/cureus.71709

**Published:** 2024-10-17

**Authors:** Mosab Hussen Mostafa Adam, Sara Bakhit, Mohamed E Ahmed, Mohamed A Almahal, Hiba A Ali, Mayada O Ahmed, Basil A Ibrahim, Fatima A Elmustafa, Sara N Ibrahim, Omer El Faroug H Salim

**Affiliations:** 1 Orthopedics, University of Khartoum, Khartoum, SDN; 2 Faculty of Medicine, University of Khartoum, Khartoum, SDN; 3 Internal medicine, University of Khartoum, Khartoum, SDN; 4 Internal Medicine, University of Khartoum, Khartoum, SDN; 5 Anatomy Department, Faculty of Medicine, University of Khartoum, Khartoum, SDN; 6 General Surgery, Soba University Hospital, Khartoum, SDN; 7 MBBS, Faculty Of Medicine, University Of Khartoum, Khartoum, SDN; 8 Internal Medicine, Ahfad University for Women, Omdurman, SDN; 9 Fellowship of the Royal College of Surgeons of Ireland (FRCSI), Faculty of Medicine, University of Khartoum, Soba Teaching Hospital, Khartoum, SDN

**Keywords:** electrolytes, fasting patients, gastrointestinal surgery, intravenous fluids, post-operative

## Abstract

Background: Postoperative fluid and electrolyte management is crucial for adequate patients’ recovery and healing. This audit aimed to assess the current practice of postoperative intravenous fluids (IV) and electrolyte administration and investigate postoperative electrolyte disturbances.

Method: This study was conducted at Soba Teaching Hospital and comprised two cycles, it examined adult patients who underwent abdominal surgery and were exclusively on postoperative intravenous fluid therapy for at least one day. Exclusions were made for certain conditions (Heart or renal failure, ICU admissions, day surgeries). Data collected from medical records including intravenous fluid types and amounts, electrolytes, and daily serum sodium and potassium levels, were compared to British Consensus Guidelines on Intravenous Fluid Therapy for Adult Surgical Patients (GIFTASUP). Following cycle one, regular educational sessions were conducted for medical staff, emphasizing the need for improved practices in postoperative care.

Results: A total of 14 patients in cycle one and 15 patients in cycle two were included. The data analysis unit was the patient-day. Patient-days refers to the total number of days patients were on IV fluids after surgery, starting from the day after the operation and excluding the day of surgery. It is calculated by adding up the number of days each patient remained exclusively on IV fluids. A total of 33 and 30 patient-days were analyzed in cycle one and cycle two respectively. The recommended daily amount of IV fluids for maintenance was given in 0.00% of patient-days in cycle one as compared to 76.7% in cycle two. Sodium and potassium doses were given within the recommended range of 0.00% and 24% patient-days respectively in cycle one. In cycle two, sodium and potassium were given according to the guidelines in 46.7% and 60% of patient-days respectively. Electrolyte disturbances occurred in 69.7% of patient-days in cycle one, decreasing to 46.7% in cycle two, primarily in the form of hyponatremia and hypokalemia.

Conclusion: This study highlighted the need for continued monitoring and emphasized the importance of adequate medical staff training about postoperative IV fluids and electrolytes.

## Introduction

Management of postoperative intravenous fluids and electrolytes is a basic and vital part of surgical care. Postoperative fluid administration aims to maintain normal fluid and electrolytes physiology by ensuring adequate fluid and electrolytes prescription as maintenance and replacing the ongoing losses [1].

Proper prescription and administration of fluids are extremely important, especially in gastrointestinal surgery where the patients are incapable of taking their needs orally. Body fluid composition may change in minutes or hours and can lead to impaired wound healing and homeostatic disturbances. Hence, adequate fluids and electrolytes management is critical to ensure sufficient tissue perfusion and normal hemodynamics to lower the incidence of homeostatic complications [2].

The majority of the intravenous fluids administered to surgical patients are given within the postoperative phase and the majority of these fluids are prescribed by the junior surgical staff [3]. Improper care and poor knowledge regarding the basics of fluids and electrolyte balance are identified causes of unfavorable outcomes in surgical patients [4].

There are multiple guidelines about postoperative intravenous fluids and electrolytes management for both maintenance and replacement, aiming to guide and improve the quality of practice regarding the prescription and administration of fluids and electrolytes [5,6]. These guidelines are closely similar and represent a suitable standard that helps in assessing the practice, putting in mind that sometimes deviations from these guidelines may be needed according to the patient’s status and local considerations such as availability, cost, and custom hospital guidelines. Despite these guidelines, the practice regarding postoperative IV fluids and electrolytes prescription and administration still varies among prescribers and is sometimes found to be improper [4].

Surgical care aims to improve the patient’s well-being and prevent adverse events afterward. The complications of improper or inadequate prescription and administration might carry a lifelong burden for the patients and affect their quality of life and most importantly their satisfaction with the provided service [2].

This audit was conducted to assess the current postoperative IV fluids and electrolytes prescriptions and administration practice and to investigate postoperative electrolyte disturbances.

## Materials and methods

Study area and design

This observational prospective audit was conducted at Soba Teaching Hospital (Khartoum-Sudan), general surgery department (Gastrointestinal unit) where the average number of admissions is 16 patients per month.

Ethical approval

This quality improvement project was reviewed and approved by the surgery department and the quality improvement center of the hospital. Informed verbal consent was obtained from all participating patients to access clinical information recorded in patients’ files.

Sampling

Total coverage of admissions meeting the inclusion criteria during the study period. Inclusion Criteria were patients aged 18 or more, who underwent gastrointestinal surgery and were exclusively on postoperative intravenous fluid therapy (Nill per mouth) for at least one day after the surgery.

Exclusion criteria included patients with heart or renal failure, those on parenteral nutrition, day-case surgery, ICU-admitted patients, and those who were hemodynamically unstable. Additionally, patients not exclusively on IV fluids, as well as those with preoperative abnormal serum sodium and potassium levels, were also excluded.

Standards and criteria

The criteria used in this audit were from The British Consensus Guidelines on Intravenous Fluid Therapy for Adult Surgical Patients (GIFTASUP) [5].

The type and amount of intravenous fluid and electrolytes administered postoperatively were compared with recommended values for weight. The standards were set at 90% by consensus (Table 1).

**Table 1:** Intravenous fluid and electrolytes prescription criteria and standards for postoperative maintenance The standards were set at 90% by consensus with the department consultants to maintain a realistic and achievable goal in a clinical setting with potential challenges, such as resource limitations and human error, especially in a context like Sudan.

Criteria	Standards
1 Patient should receive 1500–2500 ml of water per day for maintenance (interpreted by us as 25–35 ml.kg.day^-1^ of water)	90%
2 Patient should receive 50–100 mmol per day of sodium for maintenance (interpreted by us as 0.8–1.2 mmol.kg.day^-1^)	90%
3 Patient should receive 40–80 mmol per day of potassium for maintenance (interpreted by us as 0.8–1.2 mmol.kg.day^-1^)	90%

Data collection

Patient-days refers to the total number of days patients were on intravenous fluids after surgery, starting from the day after the operation and excluding the day of surgery. It is calculated by adding up the number of days each patient remained exclusively on IV fluids. Data was collected on day one, day two, and day three (as long as the patient was exclusively on intravenous fluids through these days), day one is defined as the day after the operation [7], day zero (the day of the operation) was not included as fluid-prescription for this period is often written intraoperatively by anesthetists and is not expected to reflect practice. Administration of intravenous fluid was done at 6 AM, 2 PM, and 10 PM. The data for each day was collected after the last given drip and before starting fluid administration the next day. The duration of data collection was two months for each cycle. From the medical notes, data about the age, sex, preoperative weight, and operation type was collected. Data about the type and amount of prescribed intravenous fluids for maintenance was collected from both patients’ files and nursing sheets. Any discrepancy in documentation or unrecorded data was investigated by asking the nurse in charge about the amount and type of the given fluid. Other collected data included the amount of potassium and sodium administered per kilogram body weight per day, preoperative and daily electrolytes measurements, in addition to urine output, nasogastric tube drains, stoma, and other losses per 24 hours. In case of not ordering electrolytes measurement by the doctor in charge, the data collector did that after obtaining consent from the patient and explaining the purpose of this action. Serum potassium of less than 3.5 mmol/l was identified as hypokalemia, and serum potassium of more than 5.5 mmol/l was considered hyperkalemia. Hyponatremia was considered as serum sodium of less than 135 mmol/l, and hypernatremia as serum sodium of more than 145 mmol/L. All data was collected in a personalized Word document (Data Sheet) for every patient. No patient-identifiable data was recorded and kept. All recorded data was anonymized and encrypted using commercially available software. The percentages of patient-days where the recommended daily amount of IV fluids, sodium, and potassium for maintenance were correctly administered were compared between the two cycles. The term “percentages of practice” refers to the proportion of total patient-days in which the medical staff adhered to the guidelines. The total duration of this study was six months, data collection lasted from 10/04/2022 to 12/06/2022 in cycle one, and from 15/08/2022 to 14/10/2022 in cycle two.

The intervention after cycle one consisted of regular educational sessions, providing guideline posters, equipping the internal pharmacy with recommended types of IV fluids, the ward with a weight machine, and formalizing a standardized fluid chart sheet.

Educational sessions

Two weeks after the completion of cycle one and before commencing cycle two, educational sessions, and focused group discussions were conducted at the surgery department. These sessions were organized by the audit team in liaison with the surgery department. They were conducted once per week over a period of one month lasting one hour each to ensure full coverage of all junior doctors practicing in the surgery department, therefore, the gap between these sessions and cycle two was two weeks. They focused on how to assess the patient clinically and calculate patients’ fluids & electrolytes requirements, treatment of electrolyte disturbances, and the importance of appropriate documentation.

Statistical analysis

A descriptive analysis was performed. Across the two cycles, Pearson’s correlation coefficient was used to assess the correlation between the amount of IV fluids given with patient weight as well as the correlation between patient weight and the amount of sodium and potassium administered. Fisher’s exact test was used to test statistically significant differences in terms of correct practice between cycles one and two. Pearson’s correlation coefficient value (r) and two-sided P-value were measured with 0.05 or less significance level. Statistical analysis was done using Statistical Product and Service Solutions, version 25 (SPSS 25).

## Results

There was a total of 14 patients who underwent gastrointestinal surgeries during cycle one including 11 (78.5%) males and three (21.5%) females. Mean age was 48.7 ± 19.9 (mean ± SD) years. Fifteen patients were included in cycle two, including eight (53.3%) males and seven (46.7%) females, the mean age was 56.2 ± 14.4 (mean ± SD) years. The types of performed surgeries are shown in Table 2.

**Table 2:** Type of the operations

Operation	Cycle 1 ( n=14)	Cycle 2 ( n=15)
Abdominoperineal resections	6 (42.8%)	2 (13.3%)
Whipple procedures	2 (14.3%)	2 (13.3%)
Bile duct explorations	2 (14.3%)	1 (6.7%)
Subtotal gastrectomy	2 (14.3%)	0.00%
Laparotomy for abdominal sarcoma resection	2 (14.3%)	1 (6.7%)
Open cholecystectomy	0.00%	2 (13.3%)
Anterior resections of rectal cancer	0.00%	2 (13.3%)
Liver cyst resection	0.00%	1 (6.7%)
Duodenal cancer resection	0.00%	1 (6.7%)
Gastrectomy	0.00%	1 (6.7%)
Hepaticojejunostomy	0.00%	1 (6.7%)
Right hemicolectomy	0.00%	1 (6.7%)

In cycle one, oral intake was commenced after three days in 8/14 (57.2%) patients, after two days in 3/14 (21.4%) patients, and after one day in 3/14 (21.4%) patients, amounting to a total of 33 patient-days. In cycle two, oral intake was commenced after three days in 5/15 (33.3%) patients, two days in 5/15 (33.3%) patients, and one day in 5/15 (33.3%) patients, giving a total of 30 patient-days.

**Figure 1:**
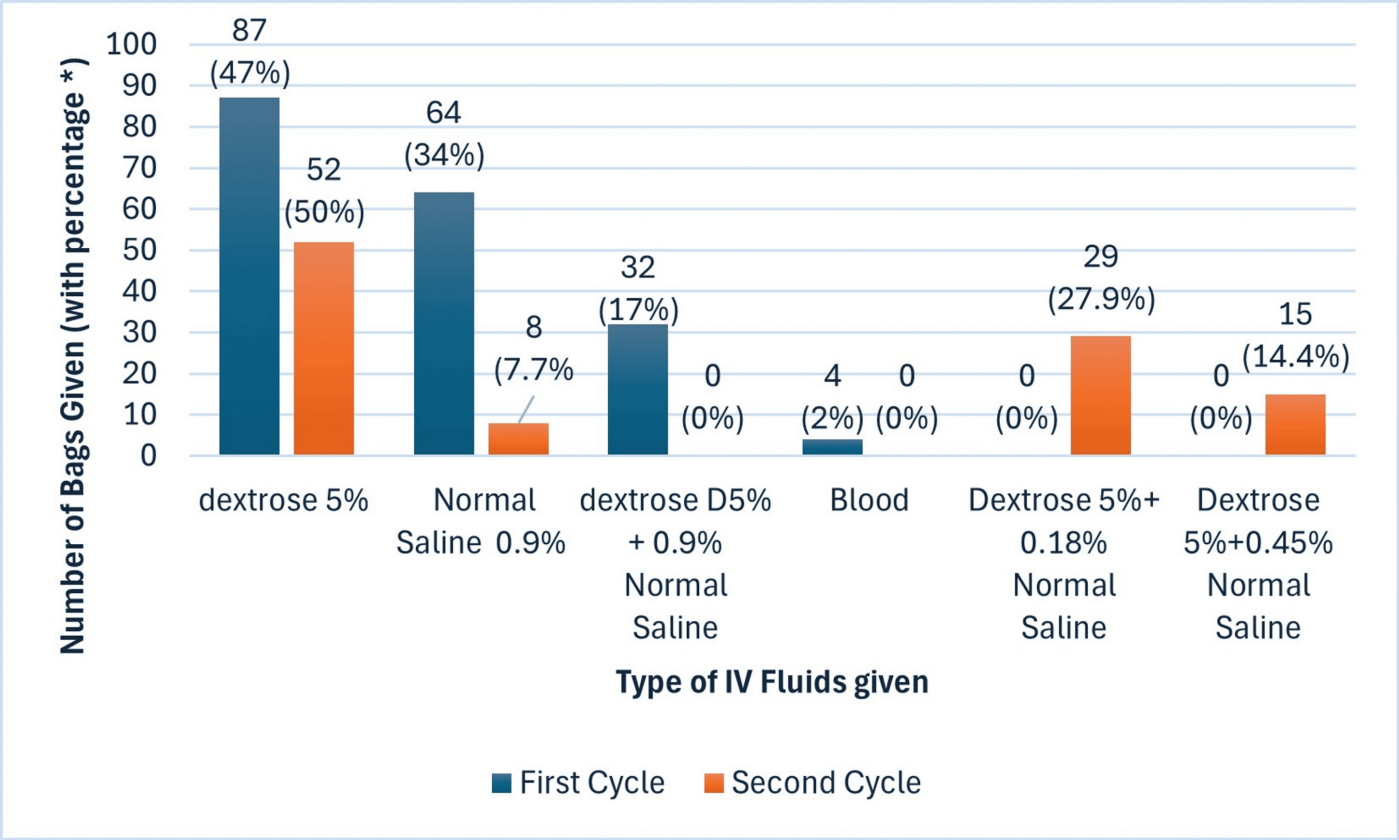
Types of IV fluids given in the two cycles *percentage is calculated out of the total number of bags given in each study cycle; 187 bags in cycle one and 104 bags in cycle two.

Dextrose (D5%) was the most prescribed IV fluids in both cycles, however, the use of hypotonic dextrose-saline increased in cycle two while prescribing 0.9 % sodium chloride decreased markedly (Figure 1).

**Figure 2:**
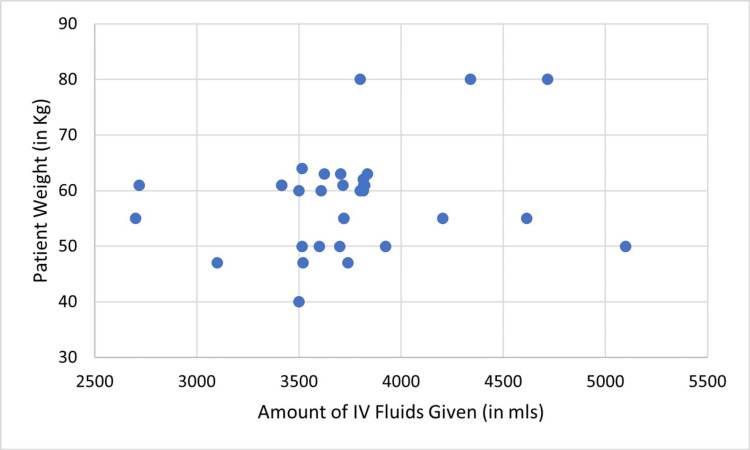
Correlation between the amount of maintenance IV fluid given and patient’s weight (cycle one), (r= 0.23, P= 0.19)

**Figure 3:**
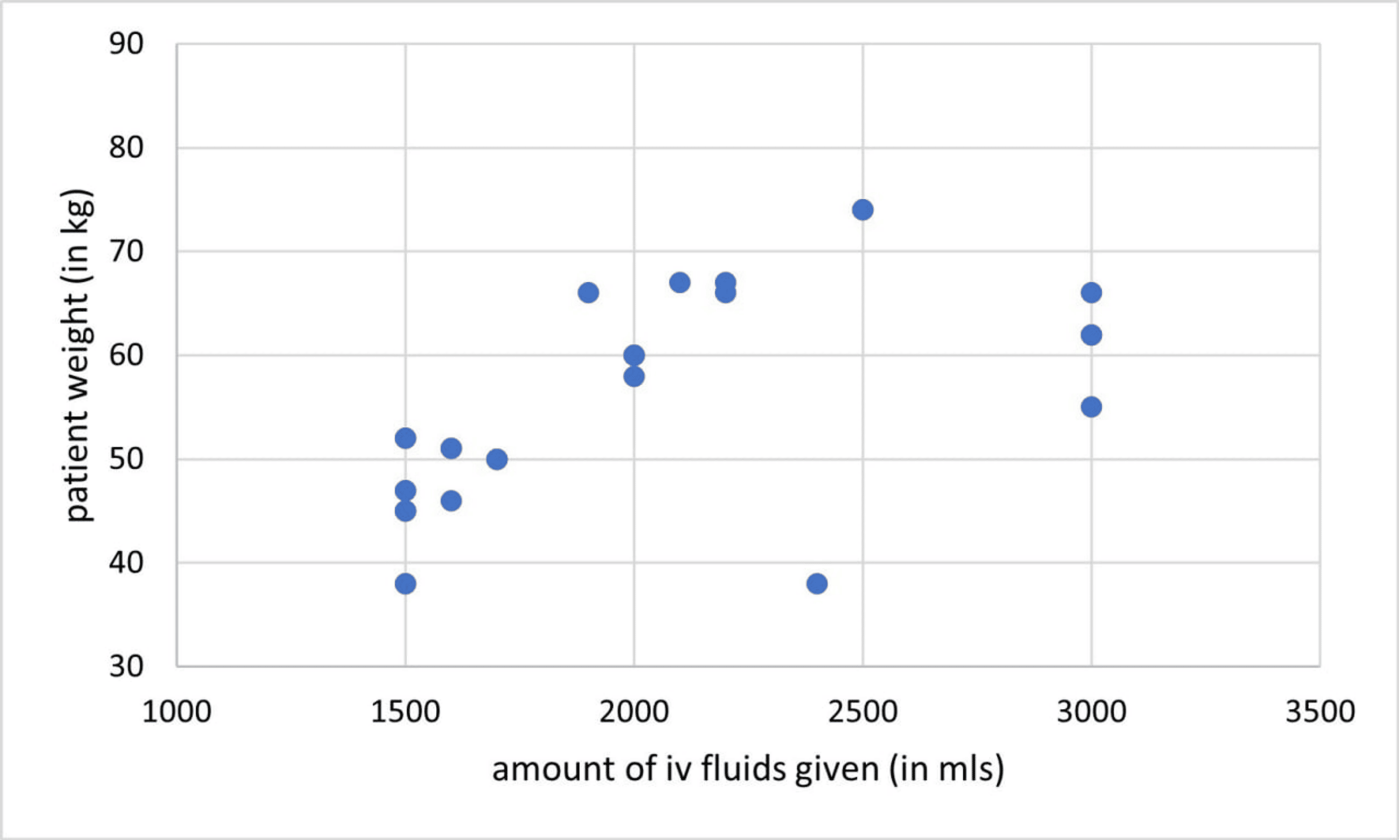
Correlation between the amount of maintenance IV fluid given and patient’s weight (cycle two), (r = 0.624, P= 0.000)

In cycle one, the recommended amount of maintenance IV fluids was given in 0/33 (0.00%) patient-days and all patients were given IV fluids above the recommended amount, additionally, no correlation was found between the fluid given for maintenance and the patient’s weight (P = 0.19, r = 0.23) (Figure 2). In cycle two, the amount of IV fluid given for maintenance was as recommended by guidelines in 23/30 (76.6%) patient-days, with a positive correlation between the given amount and the patient’s weight (r = 0.624, P= 0.000) (Figure 3).

In cycle one, extra fluid losses were poorly documented hence calculation of replacement fluid to losses was not possible except in 18/33 (54.5%) patient-days. Extra fluid losses from drains and nasogastric tubes were replaced only in one day 1/18 (5%). In cycle two, extra fluid losses were documented in 26/30 patient-days (86.7%), with losses being replaced in 19/26 (63.3%) days.

Urine output was documented in 19/33 (42.4%) days in cycle one, and there was an imbalance between fluid input and output on all these days (100%) (output= urine output + insensible loss which is 400-800 ml/day in adults without comorbidity [8]. Documentation of urine output improved to 100% in cycle two, and input-output imbalances dropped to 14/30 (46.7%) patient-days.

**Figure 4:**
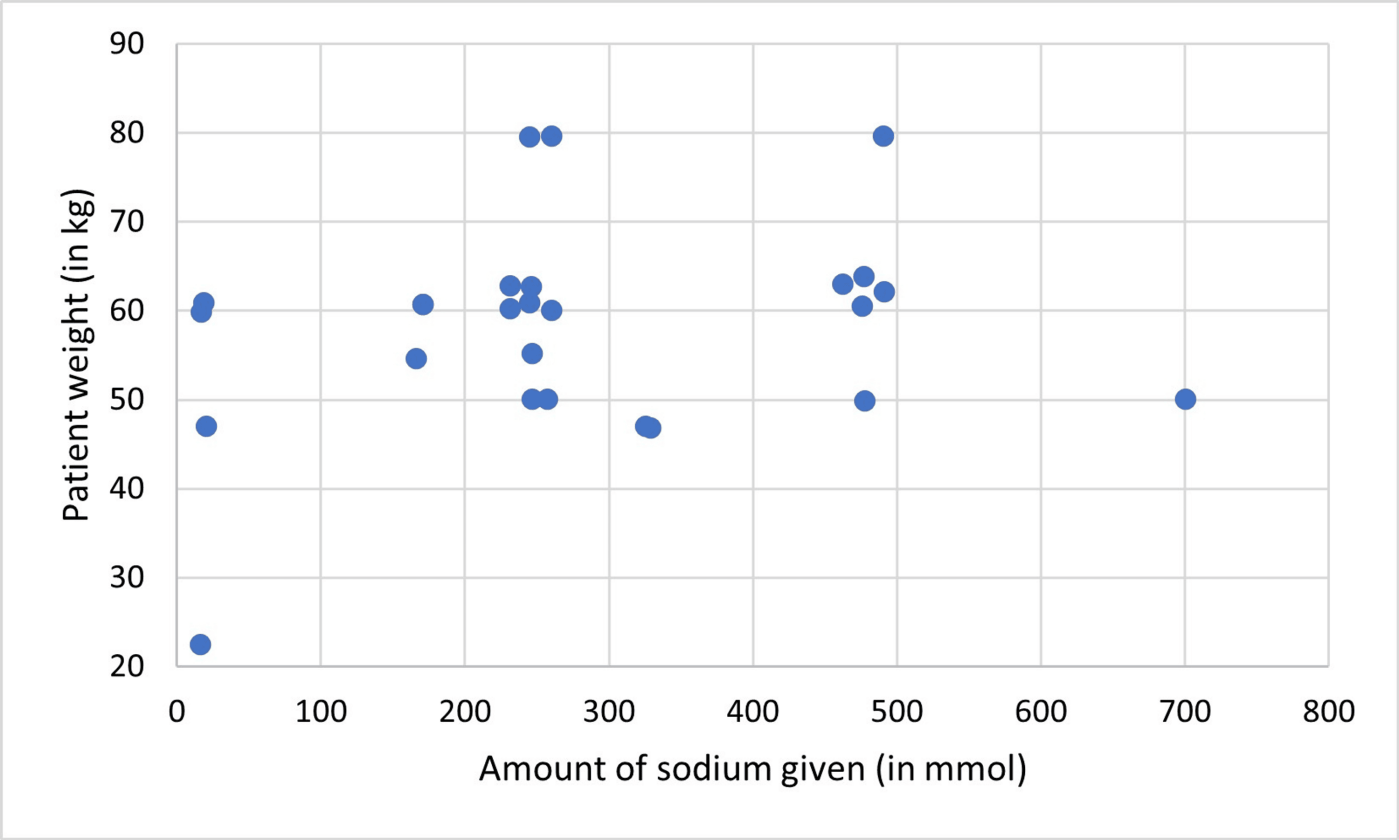
Correlation between the amount of given sodium and patients’ weight (cycle one), (r = 0.22, P = 0.21)

**Figure 5:**
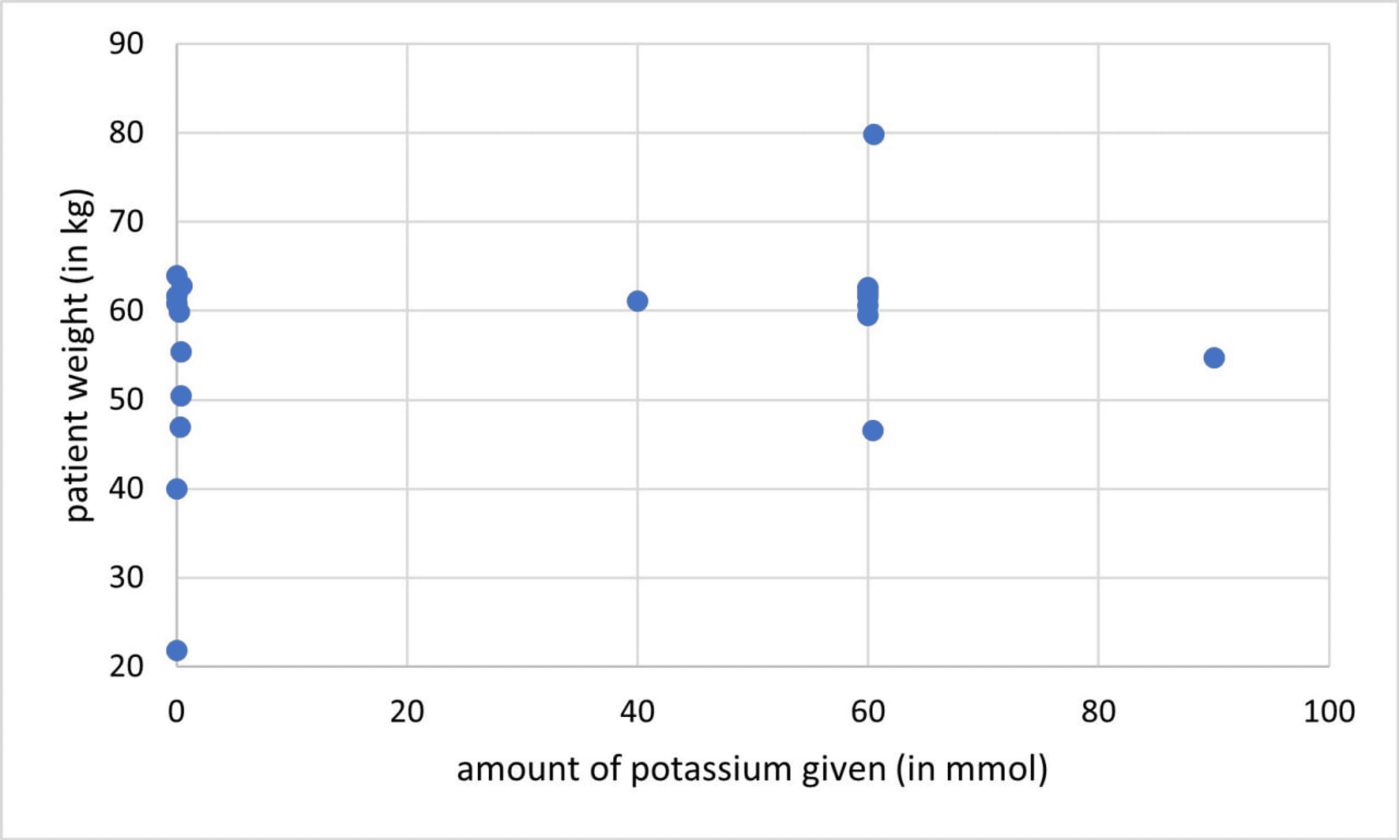
Correlation between the amount of given potassium and patient’s weight (cycle one), (r= 0.19, P= 0.30)

In cycle one, all patients were given sodium daily, however, none of them received the recommended range. Sodium was given in excess in 28/33 (85%) and below recommendations in 5/33 (15%) of patient-days. Potassium was not given in about half of patient-days 17/33 (51.5%), was administered per recommendations in 8/33 (24.2%) patient-days and in excess to or below that in 5/33 (15.2%), and 3/33 (9.1%) patient-days respectively. There was no correlation found between the patient’s weight and the amount of administered sodium (P = 0.21, r = 0.22) (Figure 4) or potassium (P = 0.30, r = 0.19) (Figure 5).

**Figure 6:**
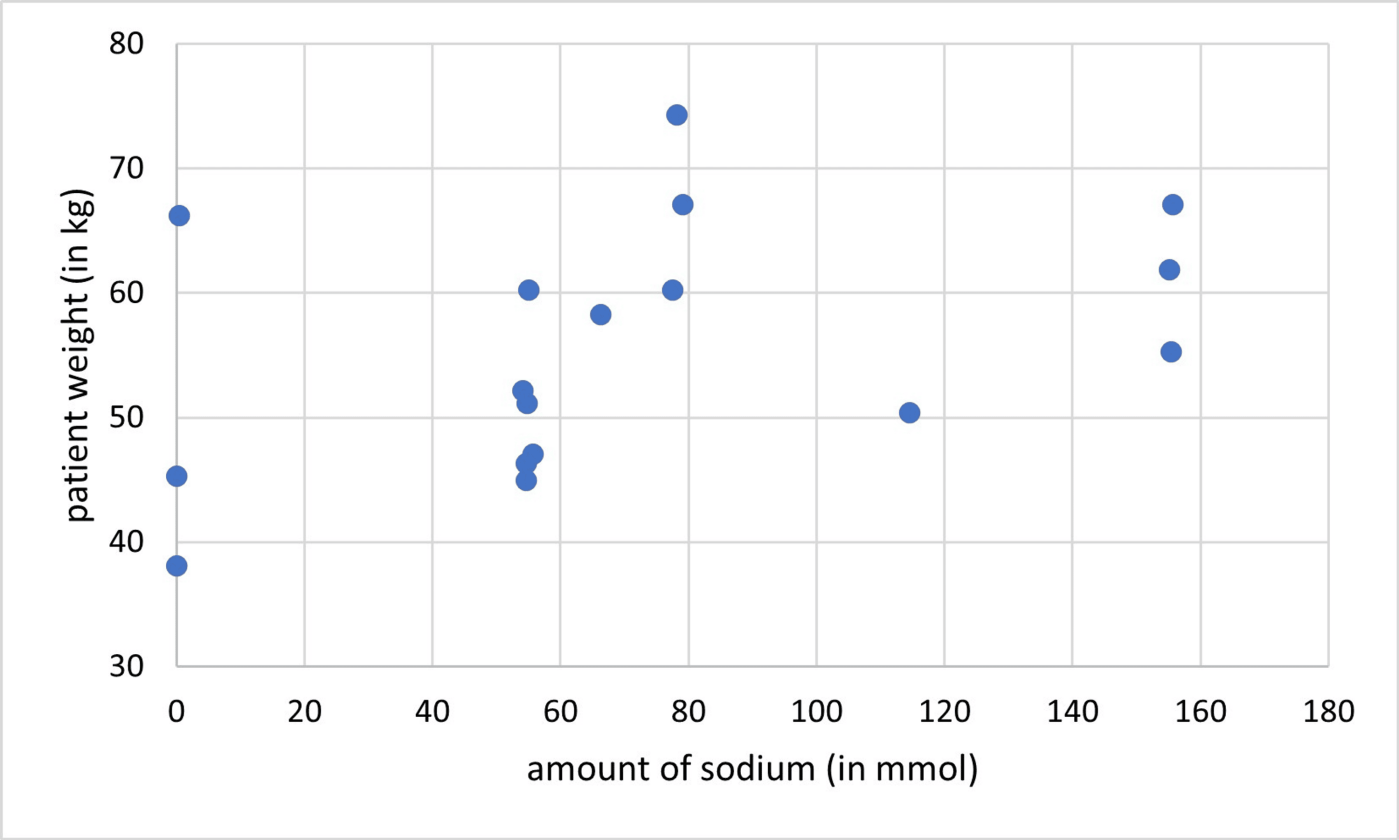
Correlation between the amount of given sodium and patients’ weight (cycle two), (r = 0.340, P= 0.066)

**Figure 7:**
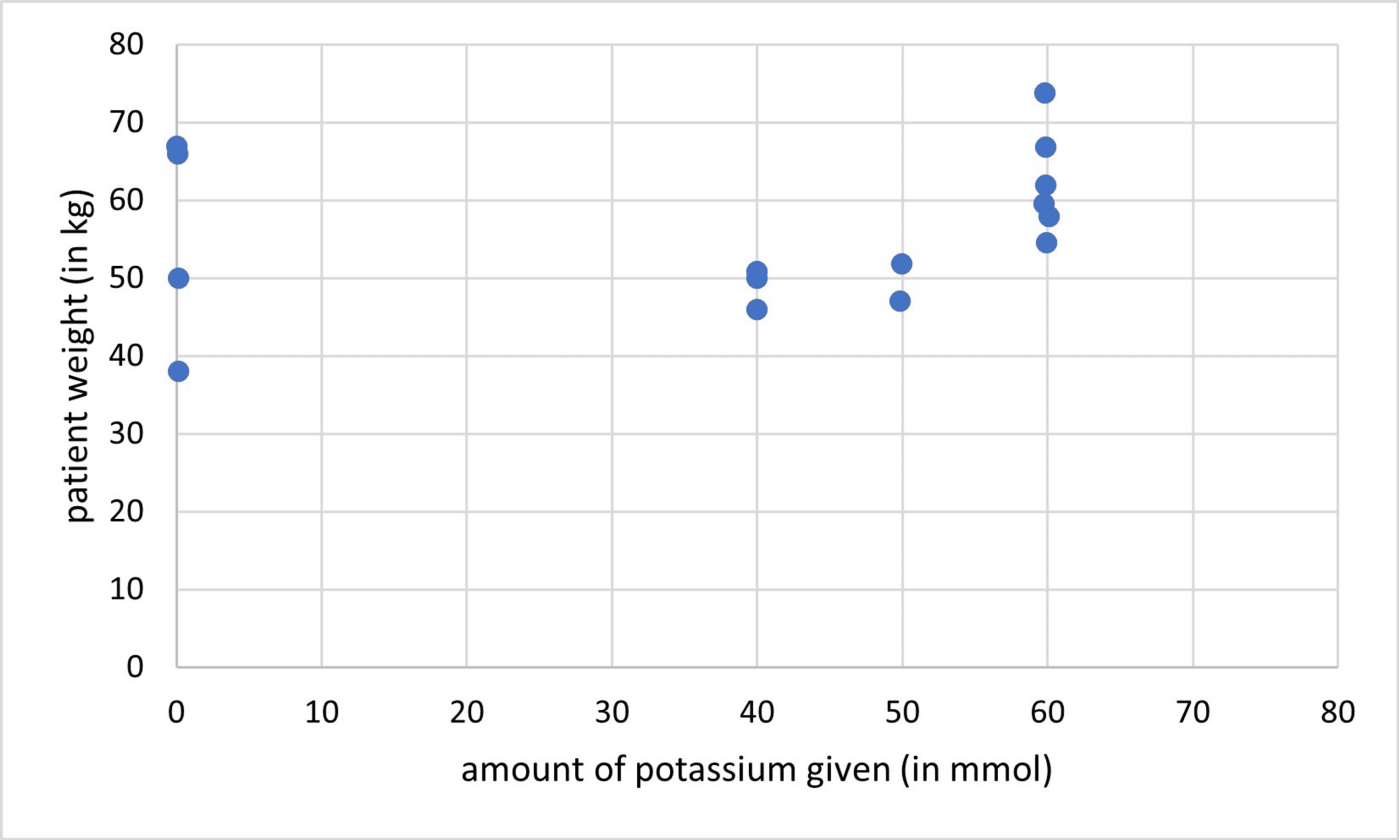
Correlation between the amount of given potassium and the patient’s weight (cycle two), (r = 0.393, P= 0.032)

In cycle two, sodium was given in 22/30 (73%) patient days. Its amount was per recommendations in 14/30 (46.7%) patient-days, and in excess in 8/30 (26.7%) patient days. Potassium, on the other hand, was administered in 18/30 (60%) patient days and it was within the recommended range in all of them. There was no correlation between sodium amount and the patient’s weight (r = 0.340, P= 0.066) (Figure 6), however, there was a positive correlation between potassium given and the patient’s weight (r = 0.393, P= 0.032) (Figure 7).

Serum electrolyte levels were requested by doctors in 17/33 (51.5%) patient-days in cycle one. However, daily electrolyte measurements were performed by the research team for the rest of the patients. Electrolyte disturbances were present in 23/33 (69.7%) patient-days in the form of hypokalemia alone in eight patient-days, hyponatremia alone in four patient-days and both hypokalemia and hyponatremia in 11 patient-days. All patients with electrolyte disturbances were receiving excess amounts of IV fluids. None of the patients developed hypernatremia or hyperkalemia. Potassium was not given at all in 10 (52.6%) out of 19 days in which hypokalemia was found. It was given per recommendation in 4/19 days (21.1%), and in excess or below recommendations in 3/19 days (15.8%) and 2/19 days (10.5%) respectively. Hyponatremia was found in 15 patient-days. Sodium was given below recommendations in 5/15 days (33.3%), and in excess in 10/15 days (66.7%). A positive correlation was found between sodium & potassium administered and their serum level (P = 0.000, r = 0.961) and (P = 0.000, r = 0.985), respectively.

In cycle two, serum electrolyte measurements were requested in 19/30 (63.3%) patient-days. After requesting electrolyte measurement for all patients, disturbances were reported in 14/30 (46.7%) patient -days. Hypokalemia alone was found in six patient-days. Potassium was not given at all in four patient-days and per recommendations on the other two days. Hyponatremia alone occurred in five patient-days, sodium was given per recommendations in four days and in excess in one day. Hypernatremia occurred in two patient-days, on which sodium was not given at all. Both hypokalemia and hyponatremia occurred in one patient-day, on which sodium and potassium were not prescribed. In this cycle, there was no significant correlation between sodium administered and its serum level (r = -0.271, P= 0.148), however, there was a positive correlation between potassium administered and its serum level (r = 0.504, P= 0.005).

In cycle one there was a gap between the standards and the practice, however, the results of cycle two showed a remarkable improvement in the practice, and the difference between cycle one and cycle two was statistically significant (p-value less than 0.05, Table 3).

**Table 3:** Comparison between the standards and percentages of practice during cycle one and cycle two The level of significance was calculated using the Fisher's Exact Test.

Criteria	Standards	Cycle one practice	Cycle two practice	p-value
1 Patient should receive 1500–2500 ml water per day for maintenance (interpreted by us as 25–30 ml.kg.day^−1^ of water)	90%	(0/33) 0.00%	(23/30) 76.7%	<0.00001
2 Patient should receive 50–100 mmol per day of sodium for maintenance (interpreted by us as 0.8–1.2 mmol.kg.day^−1^)	90%	(0/33) 0.00%	(14/30) 46.7%	0.0002
3 Patient should receive 40–80 mmol per day of potassium for maintenance (interpreted by us as 0.8–1.2 mmol.kg.day^−1^)	90%	(8/33) 24.2%	(18/30) 60%	0.0052

## Discussion

This study aimed to assess the current practice of postoperative intravenous fluids and electrolytes management after gastrointestinal surgeries and to evaluate the effectiveness of several interventions implemented to improve care.

Early return to oral intake is one of the principles of enhanced recovery after surgery protocols (ERAS). It improves patients’ comfort and gut function and limits the detrimental effects of intravenous fluid. In cycle one, more than half of the patients fasted for at least three days with no obvious indication against ERAS protocol. A study by Benjamin et al reported an early start of oral intake in most patients, however, this difference may be due to the heterogeneity among patients included in their study [1]. Raising doctors’ awareness regarding the importance of early resumption of oral intake resulted in shorter fasting durations in cycle two.

In cycle one, all patients were receiving inadequate amounts of maintenance fluid with poor adherence to the National Institute for Health and Care Excellence (NICE) and GIFTASUP guidelines. Additionally, no correlation was reported between patients’ weight and the amount of maintenance fluid given. All patients were receiving approximately the same regimen of fluid without taking into consideration variations in the patient’s weight, age, gender, volume status, and IV drugs. A study by Walsh et al reported that prescribing large amounts of IV fluids to patients was particularly done by junior house officers [3]. Benjamin et al stated that the prescription of excessive fluids was in a minority of patients with no correlation between the given quantity and the patient’s weight [1]. However, another study by Snaith et al. showed an accurate calculation of maintenance IV fluids [9].

Junior doctors often prescribe excessive amounts of IV fluids. The implementation of regular training sessions, providing guidelines posters, and formalizing a standardized fluid chart sheet have likely contributed to the substantial improvement in cycle two results. Several studies have shown similar results to cycle two. For instance, a study by Luce et al. stated that teaching and training enabled more staff to understand the importance of proper fluid management which leads to improving care and reducing inappropriate fluid administration [10]. Similarly, Gnanasampanthan et al. demonstrated that an educational intervention for junior doctors regarding appropriate fluid management led to a reduction in the volume of fluid administered to patients [11].

Complications of administering large amounts of IV fluids include sepsis, leakage, intestinal edema, and electrolyte disturbances among others [12]. On average, for patients without ongoing fluid deficits or losses, the number of fluids recommended for maintenance is 1.75 to 2.75 L/day [13]. A meta-analysis done by Vardhan and Lobo showed a reduction in complications and length of hospital stay among the patients’ group receiving 1.75 to 2.75 L/day - classified as fluid balanced - in comparison to liberal (>2.75 L/day ) and restricted (<1.75 L/day) fluid strategies [14].

Nowadays, a goal-directed strategy that focuses on unique, and individualized fluid therapy, based on fluid responsiveness (fluid need) is advocated [2]. Studies conducted on fluid therapy in hospitalized patients highlighted the importance of individualized treatment and monitoring electrolyte levels. Individualized fluid therapy based on hemodynamic monitoring, reduces the incidence of acute kidney injury in critically ill patients, improves outcomes in sepsis, and decreases the length of hospital stay [15-17].

In this study, excessive use of dextrose and 0.9% normal saline was found. Dextrose-induced hyponatremia is well-established [18]. The traditional postoperative regime of 0.9% sodium chloride and 5% dextrose increases the risk of sodium, chloride, and salt overload [19]. Sodium chloride (0.9%), even when given in reduced quantities, is associated with several detrimental effects such as hyperchloremic acidosis, reduced renal blood flow, renal failure, and increased in-hospital mortality after major abdominal surgery [20]. Hence, the NICE (2013) advocates hypotonic dextrose-saline solutions such as sodium chloride 0.18% with glucose 4% as they provide water and minimal sodium needed to meet maintenance requirements [4]. The use of hypotonic solutions increased in cycle two. Previous studies found that the use of 0.9% normal saline was common, however conducting training sessions for junior doctors helped in shifting toward more balanced electrolyte fluids like sodium chloride 0.18% with glucose 4% [21].

In cycle one, patients were given sodium more than the recommended range. Interestingly none of them suffered hypernatremia. This could be attributed to the fact that all patients received an extra amount of IV fluids. However, in a study by Benjamin Harris et al, only half the patients were given sodium more than recommended [1]. Sodium and fluid volumes are related to each other; administration of 450 mmol of sodium per day may lead to fluid overload [22]. The stress response to surgery further increases sodium retention and this can lead to serious complications like pulmonary edema and death [23].

Potassium imbalance affects heart conduction manifesting in arrhythmias. Its management postoperatively is a critical issue that demands cautious management. Fasting patients require 40 mmol slowly infused in IV fluid after the first 24 hours, keeping in mind that even small amounts of potassium in oliguric patients may be fatal [24]. In cycle one, fasting patients did not receive potassium on most days, and when given, its amount was inadequate in half the days. Unsurprisingly, hypokalemia was present in approximately two-thirds of patient-days approximately. Unfortunately, signs and symptoms of fluid or electrolyte disturbances weren’t checked; thus, we have no evidence of whether they were symptomatic or not. Our findings were similar to those reported by Rebecca et al who stated that more than half of patients didn’t receive potassium within the recommended amount [25]. Similarly, Harris et al. reported that almost all patients didn’t receive sodium and potassium which are required for daily needs [1].

Lack of knowledge among clinical practitioners regarding normal electrolyte levels, the pathophysiology behind electrolyte disturbances, and the interpretation of this knowledge into clinical practice are likely contributing factors to improper electrolyte management. Additionally, interpretation of laboratory results in light of comorbidities, patient’s clinical status, and trends in electrolyte level changes requires experience. Fluids and electrolytes are usually managed by residents with or without specialized doctors' supervision, particularly in teaching hospitals [3]. Lack of residents’ knowledge and insufficient teaching were reported in previous studies and many perioperative deaths were attributed to deficient knowledge and training of junior medicals [3,6]. This raises the need for adequate training and the establishment of clear guidelines for junior doctors to practice. In cycle two, the implementation of our action plan resulted in an improvement in care and reduction of electrolyte disturbances. For instance, potassium was administered within the recommended range in 60% of patient-days.

Interestingly in cycle two, no significant correlation was reported between given sodium and the patient’s weight. However, a positive significant correlation between given potassium and the patient’s weight was found. This necessitates advocacy for individualized treatment for optimization of fluid therapy and minimizing complications.

In terms of specific electrolyte management, it was found that the use of hypotonic fluids resulted in a lower incidence of hyponatremia in comparison to isotonic fluids in hospitalized patients [26]. Likewise, the use of balanced crystalloid solutions, such as lactated Ringer's solution, resulted in a lower incidence of acute renal injury than the use of normal saline in critically ill patients [27].

Another important finding of this audit is the lack of proper documentation of urine output and fluid losses. Increased workload and nursing staff shortage are possible factors perpetuating negligence of documentation processes. Regular training sessions about proper documentation and increasing staff numbers are highly recommended. In a previous audit done by Sansom and Duggleby, they found that after a simple intervention in the form of a lanyard card with prescription documentation advice and a prescribing algorithm, documentation of medical notes related to IV fluids prescription has improved [28]

In Sudan, there are no local clinical guidelines for the management of electrolytes and fluids. Hence there is a discrepancy in practice among practitioners. Clinical guidelines are important in making evidence-based decisions and improving clinical practice in hospitals. They improve patient care and reduce workload and clinical care costs [29]. Management based upon protocols, compared to physician-driven ones, were reported to improve the time from diagnosis to management, decrease the number of replacement episodes, attain normal serum levels, and reduce the number of unnecessary tests and taking blood samples [29,30].

This study has several strengths as it is the first study that spots light on this topic in a tropical region, it only included patients with gastrointestinal operations to reduce heterogeneity, and it included only fasting patients with no electrolyte disturbances before surgery. All these helped in focusing the assessment on the effect of the given IV fluids.

The limitations of this study were the small sample size, the inclusion of patients from a single hospital due to limited manpower and resources, and the inability to involve experienced doctors in assessing patients clinically to look for symptoms and signs of fluids and electrolyte disturbances.

## Conclusions

The results of this study highlight the need for continued monitoring and individualized treatment of fluid therapy to prevent serious complications. Additionally, there is an urge to establish local guidelines to improve practice and prevent iatrogenic fluids and electrolytes-related injuries along with ongoing training and refreshment of staff. Further studies on this topic are recommended particularly in tropical areas like Sudan.
